# Placenta Previa Major: Prenatal Diagnosis and Uterus-Preserving Surgical Management in a Case Study From the Kingdom of Bahrain

**DOI:** 10.7759/cureus.87797

**Published:** 2025-07-12

**Authors:** Reem Hasan, Nayla Bushaqer, Amala Sunder

**Affiliations:** 1 General Physician, Bahrain Defense Force Hospital, West Riffa, BHR; 2 Obstetrics and Gynaecology, Royal College of Surgeons in Ireland - Bahrain, Busaiteen, BHR; 3 Obstetrics and Gynaecology, Bahrain Defense Force Hospital, West Riffa, BHR

**Keywords:** b-lynch suture, cesarean section (cs), intraoperative hemostasis, placenta accerta spectrum, placenta previa, prenatal imaging

## Abstract

Placenta previa major is a significant concern in maternal healthcare due to its potential to cause postpartum hemorrhage (PPH), which can lead to morbidity and mortality following childbirth. PPH is generally defined as the loss of 500 ml or more of blood from the genital tract within 24 hours of the birth of a baby. PPH can result from various factors, including uterine atony, retained placental tissue, trauma, and placenta previa. Placenta previa, where the placenta is abnormally positioned over or near the cervical opening, is particularly concerning due to its significant risks for both maternal and fetal health. This case report focuses on placenta previa major as it represents one of the most severe complications in obstetrics, often leading to substantial bleeding that requires meticulous management to protect both the mother and the newborn.

This case illustrates how prenatal imaging can guide the management of suspected placenta accreta. Despite suggestive imaging features, intraoperative findings confirmed placenta previa, emphasizing the need for surgical flexibility and appropriate use of hemostatic techniques.

## Introduction

In cases involving abnormal placentation, a cesarean section is necessary following careful preoperative evaluation and preparation. Transvaginal sonography is the gold standard for assessing the degree of placenta previa, but other diagnostic tools, such as MRI, cystoscopy, and color Doppler ultrasonography, can provide additional information. However, placenta accreta is often confirmed only during or after delivery due to imaging limitations [1]. Preoperative planning should include the storage of allogenic blood, coordination with relevant departments on treatment strategies, and ensuring the availability of necessary staff.

Typical imaging features of placenta accreta include placental lacunae, loss of the hypoechoic retroplacental zone, and abnormal vascularity. However, these findings can overlap with placenta previa, particularly when the placenta is implanted in the lower uterine segment. This overlap may contribute to false-positive diagnoses, as occurred in this case [2].

The approach to managing severe postpartum hemorrhage (PPH) has evolved with the development of advanced surgical techniques. One such method is the B-Lynch compression suture, which provides a mechanical solution to control uterine bleeding by applying longitudinal compression sutures to enhance uterine contraction and achieve hemostasis. Although the B-Lynch suture is primarily effective for bleeding from the upper uterine segment, it can be used as part of a combined surgical approach to manage hemorrhage in cases of placenta previa, where bleeding predominantly originates from the lower segment. This uterus-sparing technique has gained recognition as an important option to avoid hysterectomy, especially when medical management alone is insufficient.

## Case presentation

In July 2024, a 27-year-old woman, Gravida 4, Para 3 (G4P3), was admitted for an elective lower segment caesarean section (LSCS) at 35+6 weeks of gestation. The decision for this planned high-risk C-section was based on her history of three previous uncomplicated LSCS and preliminary diagnosis of placenta accreta in the prenatal clinic.

**Figure 1:**
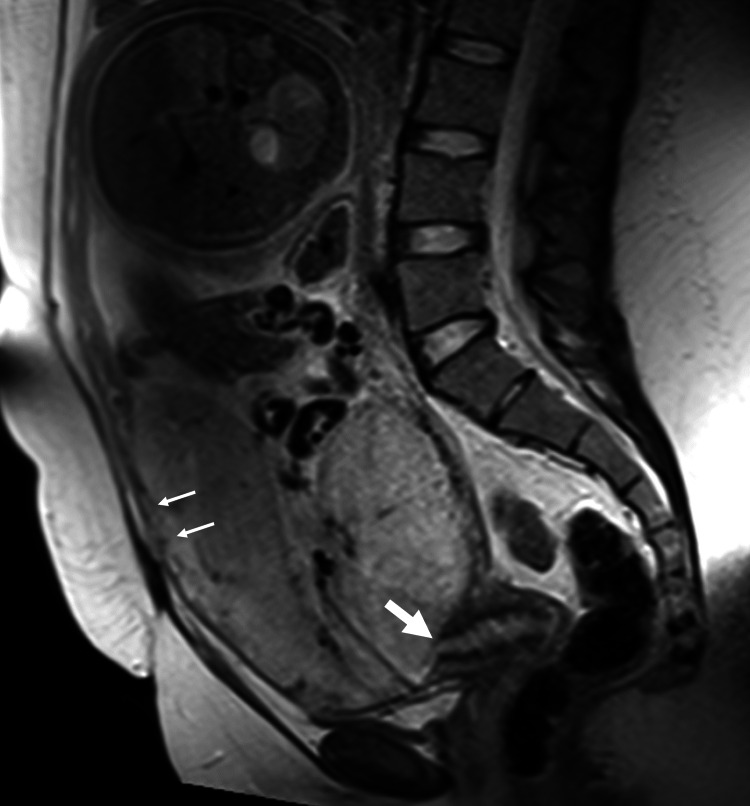
MRI Pelvis Sagittal view Placenta demonstrating increased anterior vascularity and a focal area of thinning at the myometrial interface, suggestive of placenta accreta. The internal os appears covered, but findings were more concerning for abnormal placental invasion.

**Figure 2:**
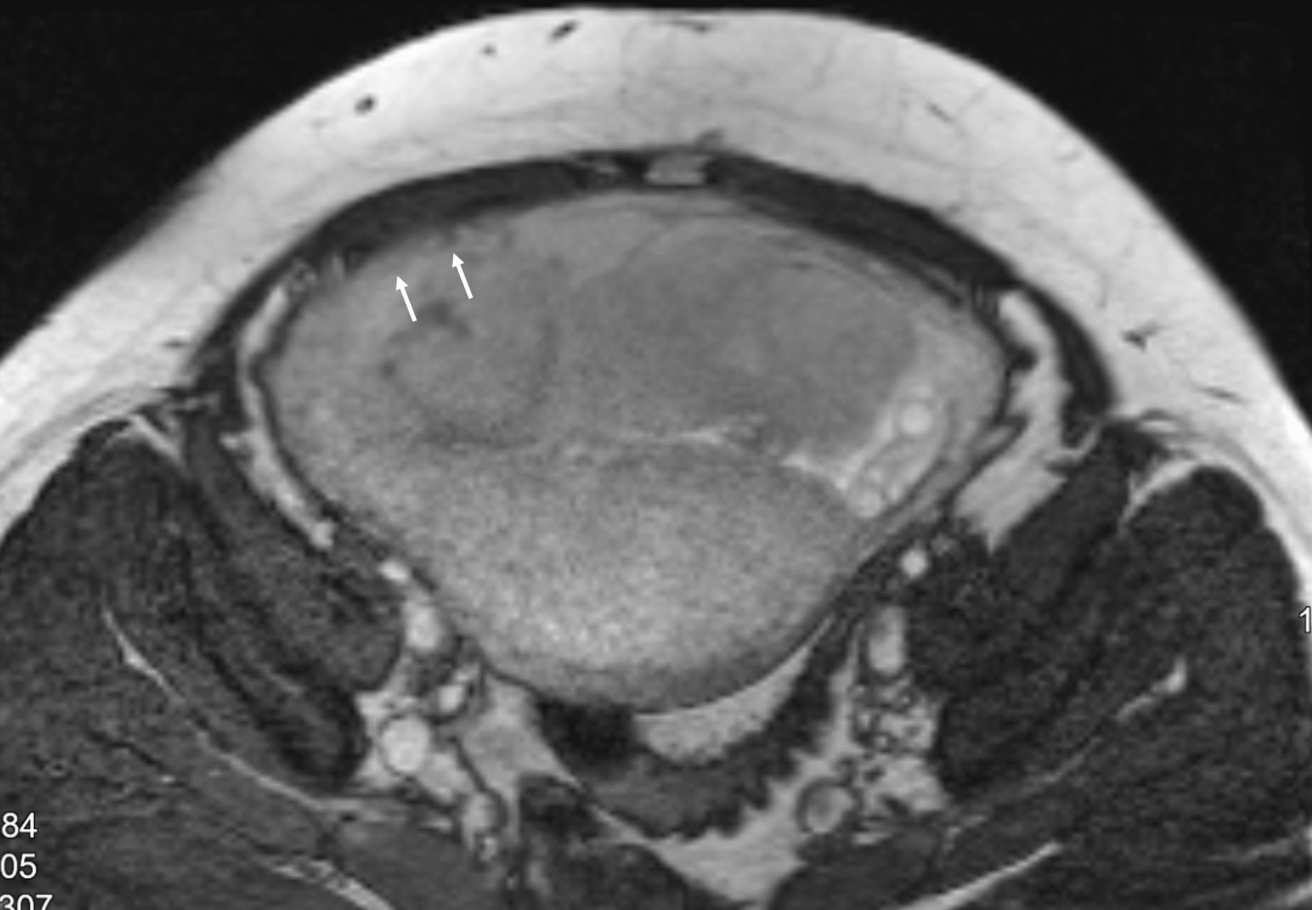
MRI Pelvis Axial view Bridging vessels and increased vascularity suggestive of abnormal placental adherence.

In the antenatal assessment, transabdominal sonography (TAS) showed a major placenta with increased vascularity at the bladder-uterus interface and bridging vessels, suggesting accreta. Pelvic MRI correlated with these findings, revealing a gravid uterus with a single fetus in cephalic presentation, a cervix measuring 4.1 cm with os closed, homogeneous placenta previa completely covering the os, and no large intraplacental bands/lakes seen. While the placenta-myometrial interface appeared intact, a focal area over the anterior lower uterine segment raised concerns about a possible placenta accreta (Figures 1, 2). Based on the previous interpretations, the patient was booked for LSCS.

The operation was performed on day two through Pfannenstiel incision. The findings included a 3 cm scar dehiscence on the right side of the uterus and a very thin, deficient lower uterine segment with placenta previa covering it. These abnormalities complicated the choice of incision site and required real-time intraoperative decision-making. Consequently, guided by preoperative ultrasonography, the uterine incision was made higher than the lower segment to minimize bleeding and avoid transecting the placenta. The amniotic sac was then opened, revealing clear amniotic fluid. The baby was delivered in cephalic presentation, followed by clamping and cutting of the umbilical cord. The baby had an appearance, pulse, grimace, activity, and respiration (APGAR) score of 9/10, weighed 3.14 kg, cried immediately after delivery, and was handed to the pediatrician. During placenta delivery, the placenta and membranes were delivered completely detaching from the uterine wall, which refuted the pre-operative suspicion of placenta accreta. Thereby, abnormal placentation was categorized upon intraoperative findings as placenta previa major.

Despite multiple hemostatic sutures, there was active bleeding from the placental bed. Therefore, vertical compression sutures (B-Lynch sutures) were applied, which successfully controlled the bleeding. The total operation time was one hour and 15 minutes, including the time taken for bilateral tubal ligation, during which the patient received two units of packed red blood cells. Estimated blood loss was approximately 1500-2000 ml, making the application of sutures timely and necessary.

Postoperatively, the patient remained vitally stable and afebrile. The uterus contracted appropriately, and the lochia was normal. A routine complete blood count was within normal limits (Table 1), indicating that no further intervention was required, and no hysterectomy was necessary.

**Table 1:** Post-Operative Complete Blood Count (CBC) and Coagulation Profile Post-Operative Laboratory Investigations: Includes hemoglobin, hematocrit, RBCs, WBCs, platelets, and coagulation parameters. Mild anemia was noted, but other values remained within normal range.

Parameter	Result	Reference Range
Red Blood Cell Parameters		
Hemoglobin (Hb)	11.4 g/dL (L)	12 - 16 g/dL
Hematocrit (Hct)	34.6 % (L)	35 - 45 %
Red Blood Cells (RBC)	3.84 × 10¹²/L	3.8 - 4.8 × 10¹²/L
Mean Corpuscular Volume (MCV)	90.1 fL	78 - 100 fL
Mean Corpuscular Hemoglobin (MCH)	29.7 pg	26 - 32 pg
Mean Corpuscular Hemoglobin Concentration (MCHC)	32.9 g/dL	31 - 36 g/dL
Red Cell Distribution Width (RDW)	12.3 %	11.5 - 16 %
White Blood Cell Parameters		
White Blood Cells (WBC)	8.66 × 10⁹/L	4 - 11 × 10⁹/L
Platelet Parameters		
Platelets (Plts)	255 × 10⁹/L	150 - 450 × 10⁹/L
Mean Platelet Volume (MPV)	10.1 fL	7.5 - 12 fL
Coagulation Parameters		
Prothrombin Time (PT)	13.4 sec	13 - 15 sec
International Normalized Ratio (INR)	0.98	0.98 - 1.13

Later, the patient was discharged after a total hospital stay of three full days, and the postoperative recovery period was uneventful in summary. Histopathological examination demonstrated a third-trimester single disc placenta weighing 635 g and measuring 17x1x2 cm with attached umbilical cord peripherally measuring 21x1 cm. Cut surface shows three blood vessels. Serial sections of the placenta show a tan, white area measuring 1x1 cm; repeat sections were taken twice from block B. In conclusion, Placenta Previa is associated with ischemic changes and infarction.

## Discussion

When planning for LSCS in patients with placental placement incompatible with vaginal birth, preoperative bedside ultrasound assessment of placental location is an essential component of guiding cesarean section surgery. This imaging modality provides critical details about the placental edge, migration, and implantation site, which are crucial for predicting delivery outcomes and minimizing the risk of intraoperative complications, such as excessive bleeding from the placental bed due to placental transection or morbidly adherent placenta [3].

Although ultrasound is a valuable diagnostic tool, its accuracy in identifying placental location can vary [4]. There is a possibility of false-positive results, where an ultrasound diagnosis of placenta accreta may not be corroborated by subsequent pathological examination and intraoperative findings, as in this case. This case illustrates a diagnostic challenge: the imaging findings were suggestive of placenta accreta, yet intraoperative inspection revealed no abnormal adherence. This discrepancy highlights the limitations of imaging modalities and reinforces the need for surgical teams to be prepared for a range of outcomes regardless of antenatal imaging. Contrary to what was anticipated in prenatal imaging, placental location falls under the category of Placenta Previa. Despite these limitations, preoperative ultrasound remains indispensable for the early detection of abnormal placentation, enabling the surgical team to prepare effectively and manage complex cases with heightened vigilance.

This case report complements the existing literature on the B-Lynch compression suture. Although the B-Lynch suture is widely used for uterine atony, it is not ideal for bleeding from the lower uterine segment, as typically occurs in placenta previa. In such scenarios, other surgical techniques are often more effective. These include transverse cervico-isthmic sutures, which target bleeding near the cervix, as well as direct figure-of-eight or interrupted sutures applied to bleeding vessels in the placental bed. Specialized lower uterine segment compression sutures, such as parallel vertical or purse-string sutures, can also provide better hemostasis. Current World Health Organization (WHO) and Royal College of Obstetricians and Gynaecologists (RCOG) guidelines emphasize individualized approaches to PPH management [5]. They highlight that compression sutures like B-Lynch are better suited for atony than placenta previa, where bleeding arises from the lower uterine segment. This further supports the exploration of alternative suturing techniques in such cases. The technique promoted hemostasis by compressing the uterus, enhancing uterine tone, making it particularly effective in cases of postpartum hemorrhage resulting from uterine atony. Compared to more radical interventions typically required for hemorrhage associated with placenta previa major or morbidly adherent placenta, such as uterine artery ligation, internal iliac artery ligation, or hysterectomy-B-Lynch sutures offer a uterus-sparing option in appropriately selected cases, with the advantages of being less invasive, easier to learn, and associated with lower postoperative morbidity. In contrast, radical surgical approaches carry significant risks, including impaired fertility, increased morbidity, and, in severe cases, maternal mortality [6].

According to WHO guidelines, the application of compression sutures should be considered following the failure of initial conservative management strategies in cases of postpartum hemorrhage, particularly due to uterine atony, and before advancing to vessel ligation or other more radical surgical interventions. However, it is important to note that compression sutures may not always prevent treatment failure or eliminate the need for more aggressive approaches-especially in scenarios such as placenta previa major, where bleeding originates from the lower uterine segment and alternative surgical measures are typically required [7].

This report is limited by its single-patient design and may not be generalizable. Additionally, intraoperative photos or surgical technique videos were not available to support documentation of findings or interventions.

## Conclusions

In this case, B-Lynch sutures provided temporary control of bleeding, although they are not ideal for lower uterine segment hemorrhage. The case underscores the importance of prenatal imaging for risk anticipation and the critical role of adaptable intraoperative planning in managing placenta previa.
